# Brain State-Dependent Closed-Loop Modulation of Paired Associative Stimulation Controlled by Sensorimotor Desynchronization

**DOI:** 10.3389/fncel.2016.00115

**Published:** 2016-05-10

**Authors:** Vladislav Royter, Alireza Gharabaghi

**Affiliations:** Division of Functional and Restorative Neurosurgery, and Centre for Integrative Neuroscience, Eberhard Karls University TuebingenTuebingen, Germany

**Keywords:** transcranial magnetic stimulation, neuromuscular electric stimulation, brain-machine interface, brain-computer interface, motor cortex plasticity, beta-band, brain state-dependent stimulation, closed-loop stimulation

## Abstract

**Background**: Pairing peripheral electrical stimulation (ES) and transcranial magnetic stimulation (TMS) increases corticospinal excitability when applied with a specific temporal pattern. When the two stimulation techniques are applied separately, motor imagery (MI)-related oscillatory modulation amplifies both ES-related cortical effects—sensorimotor event-related desynchronization (ERD), and TMS-induced peripheral responses—motor-evoked potentials (MEP). However, the influence of brain self-regulation on the associative pairing of these stimulation techniques is still unclear.

**Objective**: The aim of this pilot study was to investigate the effects of MI-related ERD during associative ES and TMS on subsequent corticospinal excitability.

**Method**: The paired application of functional electrical stimulation (FES) of the extensor digitorum communis (EDC) muscle and subsequent single-pulse TMS (110% resting motor threshold (RMT)) of the contralateral primary motor cortex (M1) was controlled by beta-band (16–22 Hz) ERD during MI of finger extension and applied within a brain-machine interface environment in six healthy subjects. Neural correlates were probed by acquiring the stimulus-response curve (SRC) of both MEP peak-to-peak amplitude and area under the curve (AUC) before and after the intervention.

**Result**: The application of approximately 150 pairs of associative FES and TMS resulted in a significant increase of MEP amplitudes *and* AUC, indicating that the induced increase of corticospinal excitability was mediated by the recruitment of additional neuronal pools. MEP increases were brain state-dependent and correlated with beta-band ERD, but not with the background EDC muscle activity; this finding was independent of the FES intensity applied.

**Conclusion**: These results could be relevant for developing closed-loop therapeutic approaches such as the application of brain state-dependent, paired associative stimulation (PAS) in the context of neurorehabilitation.

## Introduction

Paired associative stimulation (PAS) has become a widely used method for inducing motor cortex (M1) plasticity. Pairing peripheral electrical stimulation (ES) with single pulse transcranial magnetic stimulation (TMS) of the contralateral M1 modulates corticospinal excitability in accordance with the specific temporal pattern between these two cortical inputs (for review, see Carson and Kennedy, 2013).

Both cognitive factors such as attention (Stefan et al., 2004) and behavioral factors like voluntary muscle contraction (Stein et al., 2013) are known to influence the PAS effects, implying that this associative stimulation protocol is dependent on the brain state. This is further supported by the observation of preconditioning effects of other non-invasive stimulation techniques before a PAS protocol such as transcranial direct current stimulation (tDCS; Nitsche et al., 2007), repetitive transcranial magnetic stimulation (rTMS; Potter-Nerger et al., 2009), theta burst stimulation (TBS; Popa et al., 2013) or PAS itself (Müller-Dahlhaus et al., 2015). Facilitative or inhibitory tDCS for example has increased or decreased the effects of a subsequent PAS protocol, respectively (Nitsche et al., 2007). Such combined interventions may thereby have also beneficial effects on the motor training during neurorehabilitation (Celnik et al., 2009). Non-invasive stimulation protocols may though reveal a large variability of their induced effects as well (Delvendahl et al., 2012; Murase et al., 2015; Nicolo et al., 2015; Strube et al., 2015). When applying a facilitative PAS protocol to a group of 36 subjects for example, 24 subjects were identified as facilitators and 12 subjects as non-facilitators, i.e., revealing opposite effects after the same intervention (Delvendahl et al., 2012).

However, to date, the physiological evaluation of *intrinsic* brain state effects on peripheral-cortical PAS is scarce. The influence of prior muscle activity on stimulation effects and the large inter-trial variability of brain activity might be challenging for such a purpose (Hess et al., 1986; Di Lazzaro et al., 1998b; Darling et al., 2006; Mitchell et al., 2007).

Brain-machine interfaces (BMI) may help us to overcome some of these limitations by volitional modulation of brain activity during brain stimulation (Gharabaghi et al., 2014a; Gharabaghi, 2015; Kraus et al., 2016b). Feedback of motor imagery (MI)-related sensorimotor desynchronization (ERD), which resembles movement-related power changes (Miller et al., 2007), reinforces the targeted brain states without the necessity to perform actual movements (Wolpaw et al., 2002; Daly and Wolpaw, 2008; Jensen et al., 2011). Along these lines, proprioceptive feedback was recently shown to be particularly suited to facilitate MI-related brain self-regulation (Vukelić and Gharabaghi, 2015a). Functional electrical stimulation (FES) might provide such proprioceptive feedback while simultaneously serving as the peripheral constituent within a PAS paradigm. More specifically, FES could be used as the feedback modality for BMI-mediated brain self-regulation on the one hand and for afferent facilitation (AF) of subsequent TMS on the other, thus constituting a powerful link between these two interventions.

In this context, MI-related oscillatory modulation has already been shown to amplify both FES-related cortical effects—sensorimotor ERD (Reynolds et al., 2015) and TMS-induced peripheral responses—motor-evoked potentials (MEP; Takemi et al., 2013)—when these stimulation techniques are applied separately. However, the influence of MI-related oscillatory modulation, e.g., of the sensorimotor rhythm (SMR), on associative pairing of these stimulation techniques remains unclear. The SMR is modulated by thalamo-cortical and cortico-cortical interactions (Thut and Miniussi, 2009; Jensen and Mazaheri, 2010) and reflects the current brain state (Salinas and Thier, 2000; Chance et al., 2002), i.e., high and low activity indicates inhibitory and excitatory states, respectively. Fluctuations of the SMR may thus determine the brain’s responsiveness to an excitatory drive and thus at least partly account for the trial-to-trial variance of the MEP amplitude induced by TMS (Kiers et al., 1993; Thickbroom et al., 1999; Darling et al., 2006). The application of TMS during up-states of slow oscillation sleep waves (Bergmann et al., 2012) or during ERD of the SMR increases the instantaneous MEP amplitude (Takemi et al., 2013).

When brain state-dependent single pulse TMS was applied in a closed-loop paradigm controlled by SMR, increases of corticospinal excitability persisted beyond the period of stimulation (Kraus et al., 2016b). By contrast, the identical stimulation pattern, when applied independent of the brain state in the control experiment, resulted in a decrease of corticospinal excitability (Kraus et al., 2016b).

The α- and β-frequency bands of the SMR fluctuate during actual, imagined and observed movements with a highly correlated pattern, despite serving distinct functional mechanisms (van Wijk et al., 2012; Kilavik et al., 2013; Brinkman et al., 2014). Gating information by inhibiting task-irrelevant regions is attributed to α-activity (Jensen and Mazaheri, 2010), while β-activity disinhibits the sensorimotor cortex and mediates the coherent interaction with the muscles (Mima et al., 2000; Kristeva et al., 2007; van Wijk et al., 2012; Kilavik et al., 2013; Aumann and Prut, 2015; Naros and Gharabaghi, 2015). Such functional differences become particularly relevant in the context of therapeutic neurofeedback interventions (Bauer and Gharabaghi, 2015a,b; Naros and Gharabaghi, 2015). We therefore opted for brain self-regulation of β-band ERD as the physiological marker with which to modulate the effects of repetitive PAS on corticospinal excitability.

We used a BMI environment in conjunction with kinesthetic MI-related ERD in the β-band (16–22 Hz) to probe paired FES of hand opening with TMS of the contralateral M1, and tested for increases in corticospinal excitability, indexed by MEP amplitude and area under the curve (AUC).

## Materials and Methods

### Subjects

Six healthy subjects (mean age, 23.7 ± 6.6 years, range 20–37 years, 3 female) with no contraindications to TMS (Rossi et al., 2009) and no history of psychiatric or neurological disease were recruited for this study. Right-handedness was confirmed by the Edinburgh handedness inventory (Oldfield, 1971). All subjects gave their prior written informed consent to participation in the study, which had been approved by our local ethics committee. The study was carried out in accordance with the latest version of the Declaration of Helsinki. The general experimental setup for data recording, stimulation protocols and BMI setup has already been described in detail elsewhere (Gharabaghi et al., 2014a; Kraus et al., 2016a,b) and is cited here when applied in the same way.

### Recordings

#### Electromyography (EMG)

We used Ag/AgCI AmbuNeuroline 720 wet gel surface electrodes (Ambu GmbH, Germany) to record electromyography (EMG) activity from the left Extensor Digitorum Communis (EDC) muscle during the intervention. This muscle was chosen with future applications of this technique in paretic stroke patients in mind. We placed two electrodes on the muscle belly 2 cm apart from each other. After filtering between 0.16 Hz and 1 kHz, EMG was recorded with 1.1 kHz by the BrainAmp ExG Amplifier during the intervention and during the assessment of plastic changes (see below).

#### Electroencephalography (EEG)

Throughout the experiment, Ag/AgCl electrodes (BrainCap for TMS, Brainproducts GmbH, Germany) with DC amplifiers and an antialiasing filter (BrainAmp, Brainproducts GmbH, Germany) were used to record electroencephalography (EEG) signals in a 64 channel setup which complied with the international 10–20 system. For each experiment, impedances at all electrodes were set below 10 kΩ. Following digitization at 1.1 kHz rate, high-pass filtering with 0.16 Hz and low-pass filtering with 1000 Hz, the EEG signals were transferred for online analysis to Matlab, where they were later stored offline. Since it could influence electrophysiological recordings, we made every effort to remove any potential source of ambient noise from the experimental environment by turning off mobile phones, unplugging superfluous power supplies, computers etc. The effect of this procedure on, for example, the 50 Hz line noise, was verified online (Kraus et al., 2016a).

### TMS Protocol

**Figure 1:**
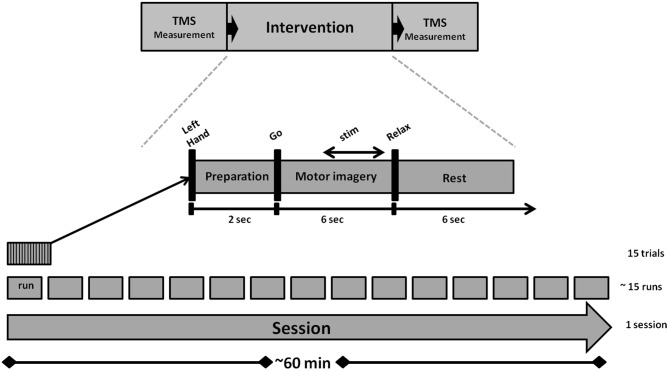
**Experimental design and structure of the study**.

We used a navigated TMS stimulator (MagPro-R30+MagOption, MagVenture, Willich, Germany) with a biphasic current waveform connected to a figure-8 MCF-B70 coil (97 mm outer diameter) to determine MEP stimulus-response curves (SRC) before and after the intervention, as well as during the intervention (Figure 1). For the coil navigation, we used frameless stereotaxy (TMS Navigator, Localite GmbH, Sankt Augustin, Germany) with a standard MNI data set (MNI ICBM152 non-linear symmetric T1 Average Brain).

Subjects were seated in a comfortable reclining chair. The representation of the left EDC in the right M1 was determined for each subject prior to the onset of the first TMS assessment. We used 40% of maximum stimulator output (MSO) at the hand knob position as the initial intensity. Whenever there was not enough initial stimulator output to elicit MEPs, we increased output in steps of 5%. We ensured that the orientation of the coil remained perpendicular to the central sulcus and defined the coil site that consistently elicited the largest MEPs as our stimulation site. The optimal coil location remained constant throughout the session (Kraus and Gharabaghi, 2015). The resting motor threshold (RMT) was then determined by the relative frequency method, i.e., by detecting the minimum stimulus intensity (in steps of 2% of MSO) that resulted in MEPs >50 μV in the peak-to-peak amplitude in at least 5 out of 10 consecutive trials (Groppa et al., 2012). We acquired the MEP stimulus-response curve (SRC) for 90, 100, 110, 120, 130, 140, 145, and 150% RMT to determine corticospinal excitability at baseline (prior to intervention) and after the intervention.

Approximately 80 stimuli were applied during each ~10-min SRC procedure. Subjects were requested to keep their muscles relaxed for the duration of all TMS measurements. We inspected the EMG data during offline analysis, discarding any trials containing muscle preactivation. Less than 1% of all trials were rejected due to contamination by muscle activity (Kraus et al., 2016a).

### Experimental Condition

An outline of the experiment is shown in Figure 1. The intervention lasted for approximately 60 min and consisted of 15 runs. Each run took approximately 2.5 min and contained 15 trials. A preparation phase of 2 s marked the onset of each trial. This was followed by a 6 s MI phase and a 6 s rest phase. The auditory cues “left hand”, “go” and “relax”—presented by a recorded female voice—marked the onset of the preparation, imagery and rest phases. In all trials, subjects performed the same kinesthetic MI task during the MI phase. They were asked to imagine and to sense the opening of their left hand, i.e., finger extension, from a first person perspective without actually moving it. Notably, they were instructed to continue with MI even when FES occurred, because MI has been shown to amplify FES-related ERD when applied concurrently and not only when triggering the peripheral stimulation (Reynolds et al., 2015).

Custom written C++ and Matlab codes were used to handle the EEG data stream and the device communication for FES of the left EDC muscle (RehaStim 2+^®^, Hasomed GmbH, Magdeburg, Germany) and TMS of the right M1 (MagPro-R30+MagOption, MagVenture, Willich, Germany). The online detection of event-related desynchronization (ERD) in the β-band (16–22 Hz) during MI phase for activating PAS was based on a BMI procedure as described in detail elsewhere (Gharabaghi et al., 2014a; Kraus et al., 2016a,b). While classical *assistive* BMI approaches choose a subject-specific frequency band and/or alpha frequency to maximize the classification accuracy e.g., between motor-imagery and rest, *rehabilitative* BMI approaches, such as the one used in this study, seek to restore the communication between cortex and periphery (Kraus et al., 2016a,b). This interaction is naturally mediated in the β-band (Mima et al., 2000; Kristeva et al., 2007; van Wijk et al., 2012; Kilavik et al., 2013). We therefore selected this frequency band to mediate the disinhibition of the sensorimotor cortex and the coherent interaction with the muscles (Naros and Gharabaghi, 2015), rather than the α-band which gates information by inhibiting task-irrelevant regions (Mazaheri and Jensen, 2010). Along these lines, self-regulation of the β-band was recently shown to be facilitated by proprioceptive rather than by visual feedback (Brauchle et al., 2015; Vukelić and Gharabaghi, 2015a). This intervention increased corticospinal excitability (Kraus et al., 2016a), activated the distributed cortical motor network (Vukelić et al., 2014; Vukelić and Gharabaghi, 2015a,b) and bridged the gap between the abilities and cortical networks of MI and motor execution (Bauer et al., 2015). We therefore opted for β-band ERD with proprioceptive feedback in this study as well.

ERD was analyzed at electrodes FC4, C4 and CP4 over the right sensorimotor area during the MI phase (McFarland et al., 2000). The threshold for initiating the PAS was calculated in every trial and defined as the difference between the mean and the standard deviation of the event-related synchronization (ERS) during rest. A linear classifier with nine features consisting of three channels (FC4, C4, and CP4) and three independent 2-Hz frequency bins for estimation of spectral power from 16 to 22 Hz was used to detect decreases in SMR power in the β-band. The spectral power was calculated with an autoregressive model order of 32 (McFarland and Wolpaw, 2008). This was fitted to the last 500 ms of the signal and updated every 40 ms. Classifier output was positive when five consecutive 40 ms epochs (i.e., 200 ms) were classified as ERD-positive. An epoch was not considered to be ERD-positive until the output of the classifier exceeded the threshold (Walter et al., 2012; Gharabaghi et al., 2014a; Kraus et al., 2016b). Stimulation did not occur whenever the threshold was not met due to ERS or when the ERD was not consistent, i.e., not long and/or strong enough. If the threshold was not met due to insufficient ERD, no PAS was initiated. Whenever the threshold was met, FES began and was followed by a TMS pulse.

FES was applied with a 1 ms pulse width and at a frequency of 100 Hz, which has been shown to optimize conditioning of subsequent TMS effects on corticospinal excitability (Mang et al., 2010). Maximum FES intensity was individually adjusted for each subject to achieve complete hand opening, resulting in a mean of 6.8 ± 1.7 mA; each FES train included a 0.5 s ramping phase and was 2.5 s long. A biphasic single TMS pulse with 110% RMT was used to stimulate the EDC “hotspot” of the right M1 50 ms following the last shock of the FES train, since this latency is particularly suited for suprathreshold proprioceptive AF of the primary motor region controlling hand and wrist muscles (Devanne et al., 2009).

### Data Analysis

Data was analyzed using Matlab R2011b (Mathworks) with a custom built code and the Fieldtrip software package (Oostenveld et al., 2011).

#### Data Pre-Processing

The analysis focused on the trials in which the subjects were able to initiate the brain-controlled paired stimulation. EEG recorded during the intervention was divided into epochs consisting of preparation (2 s), feedback (6 s) and rest (6 s). EEG trials with an amplitude range >200 μV during the non-stimulation time were automatically discarded. Additional visual inspection of the complete trial length was performed to remove any remaining artifacted trials of EEG data that had not been captured by the automated procedure. Moreover, a visual inspection of the EMG traces was performed as well to account for the muscle preactivation; trials exceeding 50 μV were excluded from the analysis. In all, about 14% of the trials were discarded. This enabled us to make an artifact-free estimation of mean β-modulation and EMG activity. Using a zero-phase lag window-based Finite Impulsive Response (FIR) filter with a filter order of 1000, the data was detrended and band-pass filtered between 2–45 Hz (EEG) and 10–250 Hz (EMG), respectively.

#### Estimation of Mean β-Modulation

For the first 1.5 s of the FES-free MI phase, a time-frequency representation of the feedback channels (FC4/C4/CP4) and the frequency range from 4 to 40 Hz (dpss taper, time step-size of 40 ms, 4 cycles adaptive window width, 0.5 Hz frequency step) was computed to estimate the mean β-modulation. Following log transformation, the event-related spectral perturbation (ERSP) was calculated for every trial, channel and frequency bin by applying the *z*-score and averaging over trials, channels and the frequencies from 16 to 22 Hz.

#### Stimulus-Response Curve (SRC)

We performed an N-way ANOVA with Time (pre- and post-intervention), Intensity (8 levels; see below) and Subject as random factors for the MEP peak-to-peak amplitude and MEP AUC. *Post hoc* testing was performed as described below for the different parameters of the SRC.

A three-parameter Boltzmann sigmoidal function was fitted using equations 1 and 2 for the peak-to-peak MEP amplitude curve and for the area under the MEP curve, respectively, of the pre and post responses of all subjects (Devanne et al., 1997; Houdayer et al., 2008; Möller et al., 2009).

(1) $$MEP(S)\;=\;MEPmax/\left(1+exp\left(\left(S_{50}-S\right)/k\right)\right)$$

(2) $$MEParea(S)\;=\;MEPmax_{area}/\left(1+exp\left(\left(S_{area\;50}-S\right)/m\right)\right)$$

In equation 1, MEP(S) represents the mean peak-to-peak MEP, and in equation 2, MEParea(S) represents the mean area under the MEP curve (AUC), as elicited by a stimulus intensity S. MEPmax and MEPmax_area_ represent the saturation amplitude of the peak-to-peak MEP amplitude and the MEP AUC, respectively. S50 and S_area_50 stand for the stimulation intensity required to obtain 50% of MEPmax and MEPmax_area_, respectively. K and m are the respective slope parameters representing the recruitment gain in the corticospinal pathway (Devanne et al., 1997).

This procedure resulted in a mean SRC of all subjects for pre- and post-intervention. A 95% confidence interval was calculated for each curve parameter and for the curves. The 95% confidence intervals of the pre- and post-intervention SRCs were then compared to each other by calculating the difference of their means and the corresponding 95% confidence intervals. Both pre- and post-intervention curve parameters were then tested for significance using the method described by Altman and Bland (2011). P values for the differences in MEPmax, MEPmax_area_, S50, S_area_50, m and k were calculated and Bonferroni corrected for multiple comparison (*α* = 0.008; Kraus et al., 2016a).

#### Correlation of SRC Change with ERD Performance

The change in the SRC (post-pre) for both the mean MEP peak-to-peak amplitude and AUC was compared to the event-related cortical power and concurrent EMG activity during FES-free MI, i.e., first 1.5 s of the task (Pearson correlation). Partial correlation analysis accounted for the subject specific FES intensity. Since we expected an inverse relationship between the ERSP and the SRC changes, we applied a one-sided *t*-test for the correlation coefficient.

## Results

**Figure 2:**
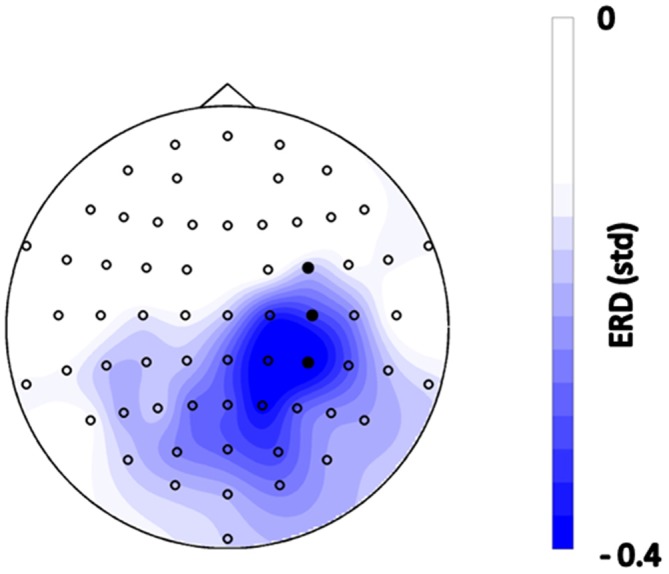
**Topographical distribution of event-related spectral perturbations (ERSPs) during the motor imagery (MI) phase before functional electrical stimulation (FES) onset, i.e., 0–1.5 s after the “Go” cue, averaged across all trials of all subjects in the frequency range of 16–22 Hz.** Blue color indicates desynchronization relative to the rest period with units in standard deviation. Filled circles indicate the feedback channels.

The subjects initiated PAS in 85.7 ± 2.3% of the trials via brain self-regulation. The average number of stimuli applied per subject was 149 ± 2.4. The EEG topography revealed lateralized sensorimotor beta-band ERD during MI in self-regulated cortex (Figure 2).

### MEP Stimulus-Response Curves

Visual inspection of the individual SRCs revealed five subjects as facilitators with increased SRCs and one subject as a non-facilitator with a strong reversed effect. The ANOVA for the facilitator group revealed a significant effect of the factor Time for both MEP peak-to-peak amplitude (*F*_(1,4)_ = 23.5; *p* < 0.01) and MEP AUC (*F*_(1,4)_ = 26.08; *p* < 0.001) and for factor Intensity for both MEP peak-to-peak amplitude (*F*_(2,4)_ = 66.61; *p* < 0.001) and MEP AUC (*F*_(7,4)_ = 63.1; *p* < 0.001). No effects were found for the interaction of Time × Intensity for MEP peak-to-peak amplitude (*F*_(7,4)_ = 0.68; *p* = 0.74) and MEP AUC (*F*_(7,4)_ = 0.36; *p* = 0.92) indicating that the MEP increased proportionally for all stimulation intensities.

**Figure 3:**
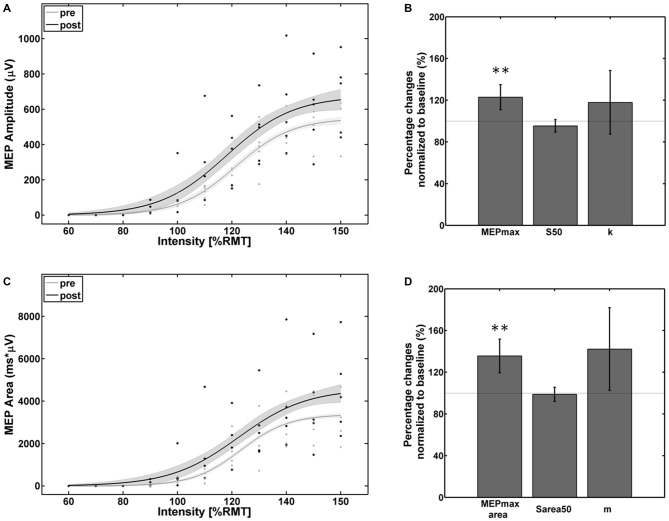
Empirical data (dots represent average of each subject) and Boltzmann fit (lines) of (A,B) the mean motor-evoked potentials (MEP) stimulus-response curves (SRCs; in μV) and (C,D) the area under the MEP curve (in μV*ms) for pre-intervention (gray) and post-intervention (black) for the subjects with increased cortico-spinal excitability. Each Boltzmann curve is paralleled by thin lines running above/below it that indicate the respective 95% confidence intervals. **p < 0.01.

The empirical data and the Boltzmann fit of the mean MEP peak-to-peak amplitude (Figure 3A) and AUC (Figure 3C) for pre- and post-intervention SRC revealed a significant increase in corticospinal excitability between 110% and 150% of RMT. In comparison to baseline, significant alterations were observed in the Boltzmann parameters MEPmax (Figure 3B) and MEPmax_area_ (Figure 3D) but not for the S50/ S50_area_ and the slope parameters (k/m): MEPmax increased to 124% (*p* < 0.001) and MEPmax_area_ increased to 136% (*p* < 0.001) of the pre-intervention baseline.

**Figure 4:**
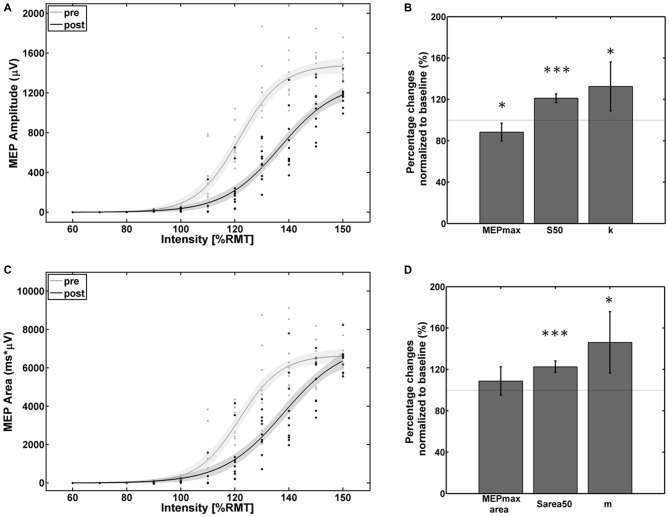
Empirical data (dots represent single pulses) and Boltzmann fit (lines) of (A,B) the mean MEP SRCs (in μV) and (C,D) the area under the MEP curve (in μV*ms) for pre-intervention (gray) and post-intervention (black) for the subject with decreased cortico-spinal excitability. Each Boltzmann curve is paralleled by thin lines running above/below it that indicate the respective 95% confidence intervals. *p < 0.05, ***p < 0.001.

The empirical data and the Boltzmann parameters for the subject with reversed effects are shown in Figure 4. A significant decrease of corticospinal excitability could be observed between 110% and 150% of RMT for the MEP peak-to-peak amplitude and between 110% and 145% of RMT for the MEP AUC. MEPmax decreased to 88% (*p* = 0.047) of the pre-intervention baseline while MEPmax_area_ showed no significant change. S50, S_area_50, k, and m increased to 121% (*p* < 0.001), 123% (*p* < 0.001), 133% (*p* < 0.05) and 146% (*p* < 0.01) of the pre-intervention baseline.

**Figure 5:**
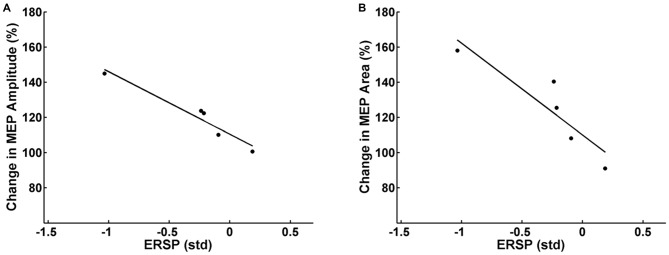
**Relation between the event-related spectral perturbation (ERSP) of the feedback frequency (16–22 Hz) during the task calculated in standard deviations (std) and the MEP peak-to-peak amplitude (A) and area under the curve (B) after the task.** Each dot represents the mean MEP and ERSP of one subject. Subjects who achieved on average more event-related desynchronization (ERD) during the task had higher average MEP amplitudes/MEP areas after the intervention.

Partial correlation analysis for the facilitator group revealed a significant inverse correlation between the mean pre-post increase of MEP peak-to-peak amplitude of each subject and the respective mean β-modulation independent of the FES intensity applied (*r* = −0.94, *p* = 0.03). In other words, the subjects who achieved on average a higher amount of ERD during the intervention underwent higher average MEP amplitude increases after the intervention (Figure 5A). Additionally, there was a significant inverse correlation (*r* = −0.91, *p* = 0.04) between mean pre-post MEP AUC increase and the mean β-modulation as well (Figure 5B) independent of FES intensity applied. No significant correlation was found between the task-related background muscle activity and the MEP peak-to-peak amplitude and AUC. Partial correlation analysis for the whole group including the non-facilitator indicated large effect sizes for the interaction between mean β-modulation and MEP peak-to-peak amplitude (*r* = −0.54, *p* = 0.17) and MEP AUC (*r* = −0.75, *p* = 0.07) as well, without reaching significance due to the small sample size.

## Discussion

In the present pilot study, we examined whether the increase of corticospinal excitability following PAS could be modulated by brain self-regulation during the intervention. As in previous applications of brain state-dependent TMS (Gharabaghi et al., 2014a; Kraus et al., 2016b), kinesthetic MI was used in this study, since the neuronal correlates activated during this task are similar to those activated during motor execution (Pfurtscheller and Neuper, 1997; Lotze et al., 1999; Neuper et al., 2005; Kaiser et al., 2011). Furthermore, the task increases corticospinal excitability (Ridding and Rothwell, 1999; Stinear and Byblow, 2004; Stinear et al., 2006; Roosink and Zijdewind, 2010) and decreases short intracortical inhibition (SICI; Abbruzzese et al., 1999) in a muscle- and time-specific way (Stinear et al., 2006). MI-related brain self-regulation was performed before and during PAS, since MI has been shown to amplify FES related ERD when applied concurrently, but not when triggering the peripheral stimulation only (Reynolds et al., 2015).

We chose high-frequency, 3-s trains of FES of the EDC muscle as the peripheral input of the pairing protocol. Our aim was to achieve complete hand opening to maximize the AF of TMS to M1, since stimulation length, intensity and frequency influence the magnitude of conditioning effects (for overview: Carson and Kennedy, 2013). If applied repetitively, such an intervention *per se* would suffice to increase corticospinal excitability, particularly when paired with suprathreshold single-pulse TMS (Devanne et al., 2009). Interestingly, however, in the present study, this PAS protocol was further modulated by MI-related β-ERD.

This intervention might be interpreted as a complementary or an additive modulation of cortical interneuronal circuits during the above combined stimulation protocol. Classical fascillatory PAS protocols with an interstimulus interval (ISI) of 25 ms (Stefan et al., 2000, 2004; Wolters et al., 2003) decrease long-interval intracortical inhibition (LICI) and long-latency afferent inhibition (LAI) without altering expressions of short-interval intracortical inhibition (SICI), intracortical fascilitation (ICF) or short-latency afferent inhibition (SAI; Carson and Kennedy, 2013). Increasing the latencies of PAS may cause a concomitant loss of SAI and induce AF of M1 circuits at an ISI of 28–35 ms (Mang et al., 2012) as well as a decrease of SICI and an increase of ICF at an ISI of 45–70 ms (Devanne et al., 2009). In this context, additional decreases of SICI (Abbruzzese et al., 1999; Takemi et al., 2013) during MI may mediate the brain state-dependent increase of corticospinal excitability in the present PAS protocol.

Neurofeedback tasks based on MI are known to be cognitively demanding (Fels et al., 2015). The occurrence of different levels of corticospinal excitability in our experiment could therefore also be related to different attentional levels (Stefan et al., 2004). Since volitional modulation of brain oscillations via MI requires by definition a certain degree of attention, its influence on the MEP changes cannot be completely ruled out. Given that the induced changes of the SRC were captured after the intervention in a state of rest, when no task was being performed, suggests however a rather specific contribution of the state of the sensorimotor loop on the PAS effects (Figures 5A,B).

Studies in which peripheral stimulation is applied with either passive movement or ES of the peroneal nerve timed to the peak negativity of the movement-related cortical potential during MI (Mrachacz-Kersting et al., 2012; Xu et al., 2014) have demonstrated an increase in corticospinal excitability that is independent of any additional cortical input, e.g., via TMS. Interestingly, when MI was performed without peripheral stimulation in these studies, no increase in corticospinal excitability was found.

Future studies will therefore need to disentangle and quantitatively compare the impact of different combinations of pairing, i.e., MI with FES, MI with TMS, and MI with FES and TMS, on corticospinal excitability. We hypothesize that in all of these approaches MI-related depolarization would stimulate the cortico-cortical connections to pyramidal neurons, albeit the exact mechanisms of TMS, FES and ERD still require clarification. In this context, decreased intracortical inhibition via motor imagery-related ERD (Takemi et al., 2013) would serve as presynaptic input for a Hebbian-like stimulation mechanism (Hebb, 1949; Kraus et al., 2016b).

The results of the present study indicate that the β-ERD brain state presents an appropriate conditioning paradigm for closed-loop approaches, including PAS, which aim to increase M1 excitability (Gharabaghi et al., 2014a; Kraus et al., 2016a,b). Moreover, these findings are in line with recent results indicating a correlation between β-modulation and MEP following a brain-robot intervention (Kraus et al., 2016a) and following a brain state-dependent TMS intervention (Kraus et al., 2016b). These findings are also in accordance with previous TMS studies which showed an inverse correlation of MEP amplitude with β-band power (Schulz et al., 2014) and an inverse correlation of intracortical inhibition with the ERD level during MI (Takemi et al., 2013). A decrease in intracortical inhibition, in turn, was shown to enhance the effectiveness of α-motor neuron recruitment, i.e., the corticospinal excitability (Devanne et al., 2002; Kouchtir-Devanne et al., 2012).

To further clarify the neurophysiological mechanisms of the increases observed in corticospinal excitability and indexed by significant changes of the peak-to-peak SRC, we analyzed changes of the area under the MEP curve. This enabled us to disentangle whether the observed increase in MEP peak-to-peak amplitude was mediated by more synchronous firing of the stimulated neuronal population (Kraus et al., 2016a), repetitive discharges of motor neurons or by the recruitment of additional neurons (Z’Graggen et al., 2005; Rösler et al., 2008). The observed increase in peak-to-peak MEP amplitudes was paralleled by a significant increase in the respective area under the MEP curve; a finding that supports the concept that the increase in corticospinal excitability is the result of the recruitment of additional neurons (Magistris et al., 1998; Rösler et al., 2002). Moreover, repetitive discharges of motor neurons cannot explain our findings, since such phenomena have been reported during additional pre-activation of the muscle only (Z’Graggen et al., 2005). Since the MEP changes in the present study were observed during rest (pre- and post-intervention), conventional explanations, i.e., relating them to the background muscle activity, a higher recruitment gain or trans-synaptic excitability of the corticospinal pathway during movement are not applicable either (Devanne et al., 1997). The increase in corticospinal excitability at the plateau of the SRC therefore probably reflects a shift of the balance between excitatory and inhibitory components of the cortical circuitry due to the additional recruitment of higher threshold corticospinal neurons (Di Lazzaro et al., 1998a, 2008, 2012). One out of six subjects in this study showed a completely different SRC after the intervention, with a significant decrease in corticospinal excitability. Although the underlying mechanism is unclear, it might be related to the recruitment of different interneuron networks, e.g., early vs. late I-waves (Hamada et al., 2013; Wiethoff et al., 2014; McCambridge et al., 2015).

With the goal of translating this approach to clinical application in stroke patients with motor deficits, some challenges remain to be considered. For example, self-regulation of beta oscillations might be limited in this patient group, since movement-related beta-ERD in the ipsilesional primary cortex is more compromised in stroke patients than in healthy controls, i.e., the more severe the patient’s motor impairment, the less beta-ERD (Rossiter et al., 2014).

Therefore, some patients may be unable to gain volitional control of beta oscillations for such an intervention via a standard EEG-based approach (Naros and Gharabaghi, 2015). This may be due to an extended cortical lesion and distorted physiology. Epidural recordings of field potentials might then facilitate the detection and neurofeedback training of this physiological target (Gharabaghi et al., 2014b). Such an approach closer to the neural signal source may also induce clinical gains after a shorter period of therapy than is usually applied with the standard EEG technique (Gharabaghi et al., 2014c) and may even serve as a bi-directional interface for concurrent brain stimulation (Gharabaghi et al., 2014d).

This pilot study has limitations, which need to be addressed in future trials. Similar to previous non-invasive stimulation studies, we also observed facilitators and non-facilitators with opposite effects following the intervention. In future, studies on the brain state-dependency of PAS need to be sufficiently powered to consider this inter-individual variability of intervention responses. Such trial will also necessitate control groups to validate the state-dependency of the present PAS effects. Such a control group may include subjects who do not perform MI or who perform MI of varying movements; other control participants may be instructed to perform event-related synchronization (ERS). Based on this pilot study, we speculate that the group without MI would show no MEP changes and that the ERS group would show a decrease of MEP after the intervention.

In conclusion, we studied the impact of the brain state on stimulation effects and showed that brain self-regulation of β-ERD during PAS modulated corticospinal excitability that persisted beyond the period of stimulation. These results could be relevant for developing closed-loop therapeutic approaches such as the application of brain state-dependent PAS in the context of neurorehabilitation.

## Author Contributions

VR: performed research, analyzed data and wrote the article. AG: designed research and wrote the article.

## Conflict of Interest Statement

The authors declare that the research was conducted in the absence of any commercial or financial relationships that could be construed as a potential conflict of interest.

## References

[B1] Abbruzzese G. Assini A. Buccolieri A. Marchese R. Trompetto C. (1999). Changes of intracortical inhibition during motor imagery in human subjects. Neurosci. Lett. 263, 113–116. 10.1016/s0304-3940(99)00120-2 10213148

[B2] Altman D. G. Bland J. M. (2011). How to obtain the P value from a confidence interval. BMJ 343:d2304. 10.1136/bmj.d2090 22803193

[B3] Aumann T. D. Prut Y. (2015). Do sensorimotor *β*-oscillations maintain muscle synergy representations in primary motor cortex? Trends Neurosci. 38, 77–85. 10.1016/j.tins.2014.12.002 25541288

[B6] Bauer R. Fels M. Vukelić M. Ziemann U. Gharabaghi A. (2015). Bridging the gap between motor imagery and motor execution with a brain-robot interface. Neuroimage 108, 319–327. 10.1016/j.neuroimage.2014.12.026 25527239

[B4] Bauer R. Gharabaghi A. (2015a). Reinforcement learning for adaptive threshold control of restorative brain-computer interfaces: a Bayesian simulation. Front. Neurosci. 9:36. 10.3389/fnins.2015.00036 25729347 PMC4325901

[B5] Bauer R. Gharabaghi A. (2015b). Estimating cognitive load during self-regulation of brain activity and neurofeedback with therapeutic brain-computer interfaces. Front. Behav. Neurosci. 9:21. 10.3389/fnbeh.2015.00021 25762908 PMC4329795

[B7] Bergmann T. O. Mölle M. Schmidt M. A. Lindner C. Marshall L. Born J.. (2012). EEG-guided transcranial magnetic stimulation reveals rapid shifts in motor cortical excitability during the human sleep slow oscillation. J. Neurosci. 32, 243–253. 10.1523/JNEUROSCI.4792-11.2012 22219286 PMC6621327

[B8] Brauchle D. Vukelić M. Bauer R. Gharabaghi A. (2015). Brain state-dependent robotic reaching movement with a multi-joint arm exoskeleton: combining brain-machine interfacing and robotic rehabilitation. Front. Hum. Neurosci. 9:564. 10.3389/fnhum.2015.00564 26528168 PMC4607784

[B9] Brinkman L. Stolk A. Dijkerman H. C. de Lange F. P. Toni I. (2014). Distinct roles for alpha- and beta-band oscillations during mental simulation of goal-directed actions. J. Neurosci. 34, 14783–14792. 10.1523/JNEUROSCI.2039-14.2014 25355230 PMC4212072

[B10] Carson R. G. Kennedy N. C. (2013). Modulation of human corticospinal excitability by paired associative stimulation. Front. Hum. Neurosci. 7:823. 10.3389/fnhum.2013.00823 24348369 PMC3847812

[B12] Celnik P. Paik N. J. Vandermeeren Y. Dimyan M. Cohen L. G. (2009). Effects of combined peripheral nerve stimulation and brain polarization on performance of a motor sequence task after chronic stroke. Stroke 40, 1764–1771. 10.1161/STROKEAHA.108.540500 19286579 PMC2692264

[B13] Chance F. S. Abbott L. F. Reyes A. D. (2002). Gain modulation from background synaptic input. Neuron 35, 773–782. 10.1016/s0896-6273(02)00820-6 12194875

[B14] Daly J. J. Wolpaw J. R. (2008). Brain-computer interfaces in neurological rehabilitation. Lancet Neurol. 7, 1032–1043. 10.1016/S1474-4422(08)70223-0 18835541

[B15] Darling W. G. Wolf S. L. Butler A. J. (2006). Variability of motor potentials evoked by transcranial magnetic stimulation depends on muscle activation. Exp. Brain Res. 174, 376–385. 10.1007/s00221-006-0468-9 16636787 PMC3582032

[B16] Delvendahl I. Jung N. H. Kuhnke N. G. Ziemann U. Mall V. (2012). Plasticity of motor threshold and motor-evoked potential amplitude. A model of intrinsic and synaptic plasticity in human motor cortex? Brain Stimul. 5, 586–593. 10.1016/j.brs.2011.11.005 22445536

[B17] Devanne H. Cohen L. G. Kouchtir-Devanne N. Capaday C. (2002). Integrated motor cortical control of task-related muscle during pointing in humans. J. Neurophysiol. 87, 3006–3017. 10.1152/jn.00990.2001 12037204

[B18] Devanne H. Degardin A. Tyvaert L. Bocquillon P. Houdayer E. Manceaux A.. (2009). Afferent-induced facilitation of primary motor cortex excitability in the region controlling hand muscles in humans. Eur. J. Neurosci. 30, 439–448. 10.1111/j.1460-9568.2009.06815.x 19686433

[B19] Devanne H. Lavoie B. A. Capaday C. (1997). Input-output properties and gain changes in the human corticospinal pathway. Exp. Brain Res. 114, 329–338. 10.1007/pl00005641 9166922

[B20] Di Lazzaro V. Oliviero A. Profice P. Saturno E. Pilato F. Insola A.. (1998a). Comparison of descending volleys evoked by transcranial magnetic and electric stimulation in conscious humans. Electroencephalogr. Clin. Neurophysiol. 109, 397–401. 10.1016/s0924-980x(98)00038-1 9851296

[B22] Di Lazzaro V. Restuccia D. Oliviero A. Profice P. Ferrara L. Insola A.. (1998b). Effects of voluntary contraction on descending volleys evoked by transcranial stimulation in conscious humans. J. Physiol. 508, 625–633. 10.1111/j.1469-7793.1998.625bq.x 9508823 PMC2230886

[B21] Di Lazzaro V. Profice P. Ranieri F. Capone F. Dileone M. Oliviero A.. (2012). I-wave origin and modulation. Brain Stimul. 5, 512–525. 10.1016/j.brs.2011.07.008 21962980

[B23] Di Lazzaro V. Ziemann U. Lemon R. N. (2008). State of the art: physiology of transcranial motor cortex stimulation. Brain Stimul. 1, 345–362. 10.1016/j.brs.2008.07.004 20633393

[B24] Fels M. Bauer R. Gharabaghi A. (2015). Predicting workload profiles of brain-robot interface and electromygraphic neurofeedback with cortical resting-state networks: personal trait or task-specific challenge? J. Neural. Eng. 12:046029. 10.1088/1741-2560/12/4/046029 26170164

[B25] Gharabaghi A. (2015). Activity-dependent brain stimulation and robot-assisted movements for use-dependent plasticity. Clin. Neurophysiol. 126, 853–854. 10.1016/j.clinph.2014.09.004 25260322

[B27] Gharabaghi A. Kraus D. Leão M. T. Spüler M. Walter A. Bogdan M.. (2014a). Coupling brain-machine interfaces with cortical stimulation for brain-state dependent stimulation: enhancing motor cortex excitability for neurorehabilitation. Front. Hum. Neurosci. 8:122. 10.3389/fnhum.2014.00122 24634650 PMC3942791

[B28] Gharabaghi A. Naros G. Khademi F. Jesser J. Spüler M. Walter A.. (2014b). Learned self-regulation of the lesioned brain with epidural electrocorticography. Front. Behav. Neurosci. 8:429. 10.3389/fnbeh.2014.00429 25538591 PMC4260503

[B26] Gharabaghi A. Naros G. Walter A. Grimm F. Schuermeyer M. Roth A. (2014c). From assistance towards restoration with an implanted brain-computer interface based on epidural electrocorticography: a single case study. Restor. Neurol. Neurosci. 32, 517–525. 10.3233/RNN-140387 25015699

[B29] Gharabaghi A. Naros G. Walter A. Roth A. Bogdan M. Rosenstiel W.. (2014d). Epidural electrocorticography of phantom hand movement following long-term upper-limb amputation. Front. Hum. Neurosci. 8:285. 10.3389/fnhum.2014.00285 24834047 PMC4018546

[B30] Groppa S. Oliviero A. Eisen A. Quartarone A. Cohen L. G. Mall V.. (2012). A practical guide to diagnostic transcranial magnetic stimulation: report of an IFCN committee. Clin. Neurophysiol. 123, 858–882. 10.1016/j.clinph.2012.01.010 22349304 PMC4890546

[B31] Hamada M. Murase N. Hasan A. Balaratnam M. Rothwell J. C. (2013). The role of interneuron networks in driving human motor cortical plasticity. Cereb. Cortex 23, 1593–1605. 10.1093/cercor/bhs147 22661405

[B32] Hebb D. O. (1949). The Organization of Behavior: A Neuropsychological Theory. New York: Wiley.

[B33] Hess C. W. Mills K. R. Murray N. M. (1986). Magnetic stimulation of the human brain: facilitation of motor responses by voluntary contraction of ipsilateral and contralateral muscles with additional observations on an amputee. Neurosci. Lett. 71, 235–240. 10.1016/0304-3940(86)90565-3 3785745

[B34] Houdayer E. Degardin A. Cassim F. Bocquillon P. Derambure P. Devanne H. (2008). The effects of low- and high-frequency repetitive TMS on the input/output properties of the human corticospinal pathway. Exp. Brain Res. 187, 207–217. 10.1007/s00221-008-1294-z 18259738

[B36] Jensen O. Bahramisharif A. Oostenveld R. Klanke S. Hadjipapas A. Okazaki Y. O.. (2011). Using brain-computer interfaces and brain-state dependent stimulation as tools in cognitive neuroscience. Front. Psychol. 2:100. 10.3389/fpsyg.2011.00100 21687463 PMC3108578

[B35] Jensen O. Mazaheri A. (2010). Shaping functional architecture by oscillatory alpha activity: gating by inhibition. Front. Hum. Neurosci. 4:186. 10.3389/fnhum.2010.00186 21119777 PMC2990626

[B37] Kaiser V. Kreilinger A. Müller-Putz G. R. Neuper C. (2011). First steps toward a motor imagery based stroke BCI: new strategy to set up a classifier. Front. Neurosci. 5:86. 10.3389/fnins.2011.00086 21779234 PMC3132635

[B38] Kiers L. Cros D. Chiappa K. H. Fang J. (1993). Variability of motor potentials evoked by transcranial magnetic stimulation. Electroencephalogr. Clin. Neurophysiol. 89, 415–423. 10.1016/0168-5597(93)90115-6 7507428

[B39] Kilavik B. E. Zaepffel M. Brovelli A. MacKay W. A. Riehle A. (2013). The ups and downs of *β* oscillations in sensorimotor cortex. Exp. Neurol. 245, 15–26. 10.1016/j.expneurol.2012.09.014 23022918

[B41] Kouchtir-Devanne N. Capaday C. Cassim F. Derambure P. Devanne H. (2012). Task-dependent changes of motor cortical network excitability during precision grip compared to isolated finger contraction. J. Neurophysiol. 107, 1522–1529. 10.1152/jn.00786.2011 22157124

[B42] Kraus D. Gharabaghi A. (2015). Projecting navigated TMS sites on the gyral anatomy decreases inter-subject variability of cortical motor maps. Brain Stimul. 8, 831–837. 10.1016/j.brs.2015.03.006 25865772

[B44] Kraus D. Naros G. Bauer R. Leão M. T. Ziemann U. Gharabaghi A. (2016a). Brain-robot interface driven plasticity: distributed modulation of corticospinal excitability. Neuroimage 125, 522–532. 10.1016/j.neuroimage.2015.09.074 26505298

[B43] Kraus D. Naros G. Bauer R. Khademi F. Leão M. T. Ziemann U.. (2016b). Brain state-dependent transcranial magnetic closed-loop stimulation controlled by sensorimotor desynchronization induces robust increase of corticospinal excitability. Brain Stimul. [Epub ahead of print]. 10.1016/j.brs.2016.02.007 26970878

[B45] Kristeva R. Patino L. Omlor W. (2007). Beta-range cortical motor spectral power and corticomuscular coherence as a mechanism for effective corticospinal interaction during steady-state motor output. Neuroimage 36, 785–792. 10.1016/j.neuroimage.2007.03.025 17493837

[B47] Lotze M. Montoya P. Erb M. Hülsmann E. Flor H. Klose U.. (1999). Activation of cortical and cerebellar motor areas during executed and imagined hand movements: an fMRI study. J. Cogn. Neurosci. 11, 491–501. 10.1162/089892999563553 10511638

[B48] Magistris M. R. Rösler K. M. Truffert A. Myers J. P. (1998). Transcranial stimulation excites virtually all motor neurons supplying the target muscle. A demonstration and a method improving the study of motor evoked potentials. Brain 121, 437–450. 10.1093/brain/121.3.437 9549520

[B49] Mang C. S. Bergquist A. J. Roshko S. M. Collins D. F. (2012). Loss of short-latency afferent inhibition and emergence of afferent facilitation following neuromuscular electrical stimulation. Neurosci. Lett. 529, 80–85. 10.1016/j.neulet.2012.08.072 22985510

[B50] Mang C. S. Lagerquist O. Collins D. F. (2010). Changes in corticospinal excitability evoked by common peroneal nerve stimulation depend on stimulation frequency. Exp. Brain Res. 203, 11–20. 10.1007/s00221-010-2202-x 20217400

[B51] Mazaheri A. Jensen O. (2010). Rhythmic pulsing: linking ongoing brain activity with evoked responses. Front. Hum. Neurosci. 4:177. 10.3389/fnhum.2010.00177 21060804 PMC2972683

[B52] McCambridge A. B. Stinear J. W. Byblow W. D. (2015). “I-wave” recruitment determines response to tDCS in the upper limb, but only so far. Brain Stimul. 8, 1124–1129. 10.1016/j.brs.2015.07.027 26294062

[B54] McFarland D. J. Miner L. A. Vaughan T. M. Wolpaw J. R. (2000). Mu and beta rhythm topographies during motor imagery and actual movements. Brain Topogr. 12, 177–186. 10.1023/A:1023437823106 10791681

[B53] McFarland D. J. Wolpaw J. R. (2008). Sensorimotor rhythm-based brain-computer interface (BCI): model order selection for autoregressive spectral analysis. J. Neural Eng. 5, 155–162. 10.1088/1741-2560/5/2/006 18430974 PMC2747265

[B55] Miller K. J. Leuthardt E. C. Schalk G. Rao R. P. N. Anderson N. R. Moran D. W.. (2007). Spectral changes in cortical surface potentials during motor movement. J. Neurosci. 27, 2424–2432. 10.1523/JNEUROSCI.3886-06.2007 17329441 PMC6673496

[B56] Mima T. Steger J. Schulman A. E. Gerloff C. Hallett M. (2000). Electroencephalographic measurement of motor cortex control of muscle activity in humans. Clin. Neurophysiol. 111, 326–337. 10.1016/s1388-2457(99)00229-1 10680569

[B58] Mitchell W. K. Baker M. R. Baker S. N. (2007). Muscle responses to transcranial stimulation in man depend on background oscillatory activity. J. Physiol. 583, 567–579. 10.1113/jphysiol.2007.134031 17627997 PMC2167351

[B59] Möller C. Arai N. Lücke J. Ziemann U. (2009). Hysteresis effects on the input-output curve of motor evoked potentials. Clin. Neurophysiol. 120, 1003–1008. 10.1016/j.clinph.2009.03.001 19329358

[B60] Mrachacz-Kersting N. Kristensen S. R. Niazi I. K. Farina D. (2012). Precise temporal association between cortical potentials evoked by motor imagination and afference induces cortical plasticity. J. Physiol. 590, 1669–1682. 10.1113/jphysiol.2011.222851 22250210 PMC3413497

[B61] Müller-Dahlhaus F. Lücke C. Lu M.-K. Arai N. Fuhl A. Herrmann E.. (2015). Augmenting LTP-like plasticity in human motor cortex by spaced paired associative stimulation. PLoS One 10:e0131020. 10.1371/journal.pone.0131020 26110758 PMC4482149

[B62] Murase N. Cengiz B. Rothwell J. C. (2015). Inter-individual variation in the after-effect of paired associative stimulation can be predicted from short-interval intracortical inhibition with the threshold tracking method. Brain Stimul. 8, 105–113. 10.1016/j.brs.2014.09.010 25444589

[B63] Naros G. Gharabaghi A. (2015). Reinforcement learning of self-regulated *β*-oscillations for motor restoration in chronic stroke. Front. Hum. Neurosci. 9:391. 10.3389/fnhum.2015.00391 26190995 PMC4490244

[B64] Neuper C. Scherer R. Reiner M. Pfurtscheller G. (2005). Imagery of motor actions: differential effects of kinesthetic and visual-motor mode of imagery in single-trial EEG. Brain Res. Cogn. Brain Res. 25, 668–677. 10.1016/j.cogbrainres.2005.08.014 16236487

[B65] Nicolo P. Ptak R. Guggisberg A. G. (2015). Variability of behavioural responses to transcranial magnetic stimulation: origins and predictors. Neuropsychologia 74, 137–144. 10.1016/j.neuropsychologia.2015.01.033 25619851

[B66] Nitsche M. A. Roth A. Kuo M.-F. Fischer A. K. Liebetanz D. Lang N.. (2007). Timing-dependent modulation of associative plasticity by general network excitability in the human motor cortex. J. Neurosci. 27, 3807–3812. 10.1523/JNEUROSCI.5348-06.2007 17409245 PMC6672399

[B67] Oldfield R. C. (1971). The assessment and analysis of handedness: the Edinburgh inventory. Neuropsychologia 9, 97–113. 10.1016/0028-3932(71)90067-4 5146491

[B68] Oostenveld R. Fries P. Maris E. Schoffelen J.-M. (2011). FieldTrip: open source software for advanced analysis of MEG, EEG and invasive electrophysiological data. Comput. Intell. Neurosci. 2011:156869. 10.1155/2011/156869 21253357 PMC3021840

[B69] Pfurtscheller G. Neuper C. (1997). Motor imagery activates primary sensorimotor area in humans. Neurosci. Lett. 239, 65–68. 10.1016/s0304-3940(97)00889-6 9469657

[B70] Popa T. Velayudhan B. Hubsch C. Pradeep S. Roze E. Vidailhet M.. (2013). Cerebellar processing of sensory inputs primes motor cortex plasticity. Cereb. Cortex 23, 305–314. 10.1093/cercor/bhs016 22351647 PMC3539453

[B71] Potter-Nerger M. Fischer S. Mastroeni C. Groppa S. Deuschl G. Volkmann J.. (2009). Inducing homeostatic-like plasticity in human motor cortex through converging corticocortical inputs. J. Neurophysiol. 102, 3180–3190. 10.1152/jn.91046.2008 19726723

[B72] Reynolds C. Osuagwu B. A. Vuckovic A. (2015). Influence of motor imagination on cortical activation during functional electrical stimulation. Clin. Neurophysiol. 126, 1360–1369. 10.1016/j.clinph.2014.10.007 25454278 PMC4493293

[B73] Ridding M. C. Rothwell J. C. (1999). Afferent input and cortical organisation: a study with magnetic stimulation. Exp. Brain Res. 126, 536–544. 10.1007/s002210050762 10422717

[B74] Roosink M. Zijdewind I. (2010). Corticospinal excitability during observation and imagery of simple and complex hand tasks: implications for motor rehabilitation. Behav. Brain Res. 213, 35–41. 10.1016/j.bbr.2010.04.027 20433871

[B75] Rösler K. M. Petrow E. Mathis J. Arányi Z. Hess C. W. Magistris M. R. (2002). Effect of discharge desynchronization on the size of motor evoked potentials: an analysis. Clin. Neurophysiol. 113, 1680–1687. 10.1016/s1388-2457(02)00263-8 12417220

[B76] Rösler K. M. Roth D. M. Magistris M. R. (2008). Trial-to-trial size variability of motor-evoked potentials. A study using the triple stimulation technique. Exp. Brain Res. 187, 51–59. 10.1007/s00221-008-1278-z 18231784

[B77] Rossi S. Hallett M. Rossini P. M. Pascual-Leone A. Safety of TMS Consensus Group. (2009). Safety, ethical considerations and application guidelines for the use of transcranial magnetic stimulation in clinical practice and research. Clin. Neurophysiol. 120, 2008–2039. 10.1016/j.clinph.2009.08.016 19833552 PMC3260536

[B78] Rossiter H. E. Boudrias M. H. Ward N. S. (2014). Do movement-related beta oscillations change after stroke? J. Neurophysiol. 112, 2053–2058. 10.1152/jn.00345.2014 25080568 PMC4274928

[B79] Salinas E. Thier P. (2000). Gain modulation: a major computational principle of the central nervous system. Neuron 27, 15–21. 10.1016/S0896-6273(00)00004-0 10939327

[B80] Schulz H. Ubelacker T. Keil J. Müller N. Weisz N. (2014). Now I am ready-now i am not: the influence of pre-TMS oscillations and corticomuscular coherence on motor-evoked potentials. Cereb. Cortex 24, 1708–1719. 10.1093/cercor/bht024 23395847

[B81] Stefan K. Kunesch E. Cohen L. G. Benecke R. Classen J. (2000). Induction of plasticity in the human motor cortex by paired associative stimulation. Brain 123, 572–584. 10.1093/brain/123.3.572 10686179

[B82] Stefan K. Wycislo M. Classen J. (2004). Modulation of associative human motor cortical plasticity by attention. J. Neurophysiol. 92, 66–72. 10.1152/jn.00383.2003 14724259

[B83] Stein R. B. Everaert D. G. Roy F. D. Chong S. Soleimani M. (2013). Facilitation of corticospinal connections in able-bodied people and people with central nervoussystem disorders using eight interventions. J. Clin. Neurophysiol. 30, 66–78. 10.1097/WNP.0b013e31827ed6bd 23377445

[B84] Stinear C. M. Byblow W. D. (2004). Modulation of corticospinal excitability and intracortical inhibition during motor imagery is task-dependent. Exp. Brain Res. 157, 351–358. 10.1007/s00221-004-1851-z 14997259

[B85] Stinear C. M. Byblow W. D. Steyvers M. Levin O. Swinnen S. P. (2006). Kinesthetic, but not visual, motor imagery modulates corticomotor excitability. Exp. Brain Res. 168, 157–164. 10.1007/s00221-005-0078-y 16078024

[B86] Strube W. Bunse T. Malchow B. Hasan A. (2015). Efficacy and interindividual variability in motor-cortex plasticity following anodal tDCS and paired-associative stimulation. Neural Plast. 2015, 1–10. 10.1155/2015/530423 PMC4381571 25866683

[B87] Takemi M. Masakado Y. Liu M. Ushiba J. (2013). Event-related desynchronization reflects downregulation of intracortical inhibition in human primary motor cortex. J. Neurophysiol. 110, 1158–1166. 10.1152/jn.01092.2012 23761697

[B88] Thickbroom G. W. Byrnes M. L. Mastaglia F. L. (1999). A model of the effect of MEP amplitude variation on the accuracy of TMS mapping. Clin. Neurophysiol. 110, 941–943. 10.1016/s1388-2457(98)00080-7 10400209

[B89] Thut G. Miniussi C. (2009). New insights into rhythmic brain activity from TMS-EEG studies. Trends Cogn. Sci. 13, 182–189. 10.1016/j.tics.2009.01.004 19286414

[B90] van Wijk B. C. M. Beek P. J. Daffertshofer A. (2012). Neural synchrony within the motor system: what have we learned so far? Front. Hum. Neurosci. 6:252. 10.3389/fnhum.2012.00252 22969718 PMC3432872

[B93] Vukelić M. Bauer R. Naros G. Naros I. Braun C. Gharabaghi A. (2014). Lateralized alpha-band cortical networks regulate volitional modulation of beta-band sensorimotor oscillations. Neuroimage 87, 147–153. 10.1016/j.neuroimage.2013.10.003 24121086

[B91] Vukelić M. Gharabaghi A. (2015a). Oscillatory entrainment of the motor cortical network during motor imagery is modulated by the feedback modality. Neuroimage 111, 1–11. 10.1016/j.neuroimage.2015.01.058 25665968

[B92] Vukelić M. Gharabaghi A. (2015b). Self-regulation of circumscribed brain activity modulates spatially selective and frequency specific connectivity of distributed resting state networks. Front. Behav. Neurosci. 9:181. 10.3389/fnbeh.2015.00181 26236207 PMC4500921

[B94] Walter A. Ramos Murguialday A. Spüler M. Naros G. Leão M. T. Gharabaghi A.. (2012). Coupling BCI and cortical stimulation for brain-state-dependent stimulation: methods for spectral estimation in the presence of stimulation after-effects. Front. Neural Circuits 6:87. 10.3389/fncir.2012.00087 23162436 PMC3499764

[B95] Wiethoff S. Hamada M. Rothwell J. C. (2014). Variability in response to transcranial direct current stimulation of the motor cortex. Brain Stimul. 7, 468–475. 10.1016/j.brs.2014.02.003 24630848

[B96] Wolpaw J. R. Birbaumer N. McFarland D. J. Pfurtscheller G. Vaughan T. M. (2002). Brain-computer interfaces for communication and control. Clin. Neurophysiol. 113, 767–791. 10.1016/S1388-2457(02)00057-3 12048038

[B97] Wolters A. Sandbrink F. Schlottmann A. Kunesch E. Stefan K. Cohen L. G.. (2003). A temporally asymmetric Hebbian rule governing plasticity in the human motor cortex. J. Neurophysiol. 89, 2339–2345. 10.1152/jn.00900.2002 12612033

[B98] Xu R. Jiang N. Mrachacz-Kersting N. Lin C. Asín Prieto G. Moreno J. C.. (2014). A closed-loop brain-computer interface triggering an active ankle-foot orthosis for inducing cortical neural plasticity. IEEE Trans. Biomed. Eng. 61, 2092–2101. 10.1109/TBME.2014.2313867 24686231

[B99] Z’Graggen W. J. Humm A. M. Durisch N. Magistris M. R. Rösler K. M. (2005). Repetitive spinal motor neuron discharges following single transcranial magnetic stimuli: a quantitative study. Clin. Neurophysiol. 116, 1628–1637. 10.1016/j.clinph.2005.03.012 15908271

